# Comparison of robotic-assisted total knee arthroplasty: an updated systematic review and meta-analysis

**DOI:** 10.1007/s11701-024-02045-y

**Published:** 2024-07-25

**Authors:** Xinyu Fu, Yiming She, Guangwen Jin, Chengri Liu, Ze Liu, Wei Li, Ri Jin

**Affiliations:** 1 Department of Orthopedics, Yanbian University Hospital, 1327 Juzi Street, Yanji Jilin, 133002 China; 2 Department of Nephrology, Yanbian University Hospital, Yanji, Jilin, 133002 China

**Keywords:** Robot assisted, Manual, Total knee arthroplasty, Knee

## Abstract

This study was conducted to compare the changes in different clinical scores and imaging indexes of patients who underwent robot-assisted total knee arthroplasty (RA-TKA) and manual total knee arthroplasty (M-TKA). PubMed, Web of Science, Cochrane Library and Embase were searched according to PRISMA guidelines in June 2024. Search terms included “robot-assisted”, “manual” and “total knee arthroplasty”. Outcome indicators included American Knee Society Score (KSS), Western Ontario McMaster Universities Osteoarthritis Index (WOMAC), Oxford Knee Score (OKS), range of motion (ROM), Hospital for Special Surgery (HSS) score, Forgotten Joint Score (FJS), 36-Item Short Form Health Survey (SF-36), operation duration (min), intraoperative blood loss (ml), pain score, patient’s satisfaction scores, hip–knee–ankle (HKA) angle, frontal femoral component angle, frontal tibia component angle, lateral femoral component angle and lateral tibia component angle. A total of 1,033 articles were obtained after removing duplicates, and 12 studies involving 2,863 patients (1,449 RA-TKAs and 1,414 M-TKAs) were finally meta-analyzed (22–32). The baseline data of both groups were similar in all results. Meta-analysis suggested a better performance of the RA-TKA group than the M-TKA group regarding the HKA angle. The manual TKA reduced the operation time and significantly improved the range of motion. The results of > 6 months follow-up showed that M-TKA was better than RA-TKA in terms of KSS score and WOMAC. Compared with M-TKA, RA-TKA can produce more accurate prosthetic alignment, but it does not lead to better clinical results. Orthopedic surgeons should choose between two surgical procedures according to their own experience and patients’ characteristics.

**Supplementary Information:**

The online version contains supplementary material available at 10.1007/s11701-024-02045-y.

## Introduction

Total knee arthroplasty (TKA) is highly effective for treating advanced knee osteoarthritis [1]. Despite its great success and rapid development over the past two decades, 20% of patients experience unsatisfactory clinical outcomes after surgery [2, 3]. The precise positioning of components and alignment of the limb are critical factors influencing patient satisfaction and functional results following TKA [4]. In practice, achieving these standards manually can be very challenging for surgeons. The development of orthopedic robots has facilitated the widespread adoption of robot-assisted total knee replacement systems in clinical settings [5–8]. Many reports have indicated that robotic-assisted total knee arthroplasty (RA-TKA) enables more accurate bone cutting and implant placement and achieves balanced extension and flexion gaps tailored to the patient's skeletal anatomy and natural ligament balance, reducing the probability of detrimental stress and wear [9, 10]. The approach can theoretically promise more significant improvements in clinical outcomes. However, controversy persists regarding whether RA-TKA yields superior functional and clinical outcomes compared to manual TKA (M-TKA). Multiple studies report no statistically significant difference in clinical outcomes, despite favorable radiological outcomes during follow-up visits [11–13]. Based on that, many scholars have disputed its clinical significance. Therefore, we collected relevant articles and performed a meta-analysis. The study aims to compare the clinical outcomes and radiological results of RA-TKA and M-TKA through the analysis of relevant studies, thus providing a basis for physicians’ decision-making processes. The hypothesis of this study is that RA-TKA yields significantly superior outcomes than M-TKA, both clinically and radiologically.

## Materials and methods

### Literature retrieval

This study complied with the standards for Preferred Reporting Items for Systematic Reviews and Meta-Analysis (PRISMA 2020) [14] and registered in PROSPERO prospectively (CRD420234731153). Articles published in English were systematically retrieved from PubMed, Web of Science, Cochrane Library and Embase up to June 1, 2024 to comprehensively compare the efficacy and/or safety of RA-TKA and M-TKA in treating knee osteoarthritis. The following terms were searched in the database: "robot-assisted", "robotic-assisted", "robot", "robotic", “Arthroplasty”, "Knee Replacement Arthroplasties", "Robotic Assisted Surgery" and "Total Knee Arthroplasty” (Table S1). Two authors independently and impartially examined the articles that met the inclusion criteria according to the search strategy, performed data extraction and manually examined the reference list of all included studies.

### Inclusion and exclusion criteria

Studies that contained the following features were included: 1. studies with a randomized control, cohort or case–control design; 2. patients with end-stage knee osteoarthritis; 3. studies that compared RA-TKA and M-TKA; 4. evaluations including no less than one of the following indicators: American Knee Society Score (KSS), Western Ontario McMaster Universities Osteoarthritis Index (WOMAC), Oxford Knee Score (OKS), joint range of motion (ROM), 36-Item Short Form Health Survey (SF-36) score, Hospital for Special Surgery (HSS) score, Forgotten Joint Score (FJS), pain score, patient satisfaction score, operation length (min), intraoperative blood loss (ml), hip–knee–ankle (HKA) angle, frontal femoral component angle, frontal tibia component angle, lateral femoral component angle and lateral tibia component angle; 5. articles containing enough data for calculating odds ratio (OR) or weighted mean difference (WMD).

Studies were excluded if they were reviews, letters, comments, case reports, abstracts for conference presentation, articles on pediatrics and unpublished articles. We included studies on patients who underwent unilateral RA-TKA or M-TKA, and also excluded studies involving single-compartment knee arthroplasty.

### Data extraction

Data was extracted systematically and independently by two investigators (Fu Xinyu and She Yiming). Disagreements were finally resolved by the third researcher (Jin Ri). We extracted the data on first author and publication year of the article, research duration, research country, research design, sample size, patient’s age and body mass index (BMI), follow-up time and interventions. Continuous variables in the included studies presented as the median and interquartile range (IQR) or range were calculated to obtain the mean ± standard deviation using verified mathematical methods [15, 16]. For studies with missing or unreported data, the corresponding author was contacted to request for complete (if any) data.

### Quality assessment

Randomized controlled trials (RCTs) as well as cohort studies were evaluated, respectively, using the Cochrane Quality Assessment Scale and Newcastle–Ottawa Scale (NOS) [17]. The scale mainly includes three dimensions: subject selection, comparability between groups and measurement of results. Studies were given a score from 0 to 9, with a score of 7–9 representing high quality [18]. Research quality and level of evidence were separately reviewed by two researchers, and differences were handled via discussion.

### Statistical analysis

Evidence synthesis was conducted using Review Manager 5.4 (Cochrane Collaboration, Oxford, UK). Weighted mean difference (WMD) and risk ratio (OR) were adopted to assess continuous and binary variables, respectively. Indicators were all presented using 95% confidence interval (CI). Then heterogeneity (Cochran's Q) and inconsistency index (*I*^2^) of all studies were assessed by the Chi-square (X^2^) test [19]. A *p* value for the χ 2 test beneath 0.05 or *I*^2^ over 50% was considered as remarkable heterogeneity. If there was remarkable heterogeneity, a random effects model was employed to approximate the pooled WMD or OR. If not, the fixed effects model was utilized. To assess the influence of the eligible studies on the pooled results containing remarkable heterogeneity, one-way sensitivity analysis was also performed. The funnel plot was produced using Review Manager 5.4 (Cochrane Collaboration, Oxford, UK). The results involving ≥ three studies were tested by the Egger’s regression test in Stata 15.0 (Stata Corp, College Station, TX, USA) [20], and the publication bias was visually assessed. A *p* value of lower than 0.05 was deemed to have statistical significance.

## Results

### Literature retrieval and study characteristics

**Fig. 1:**
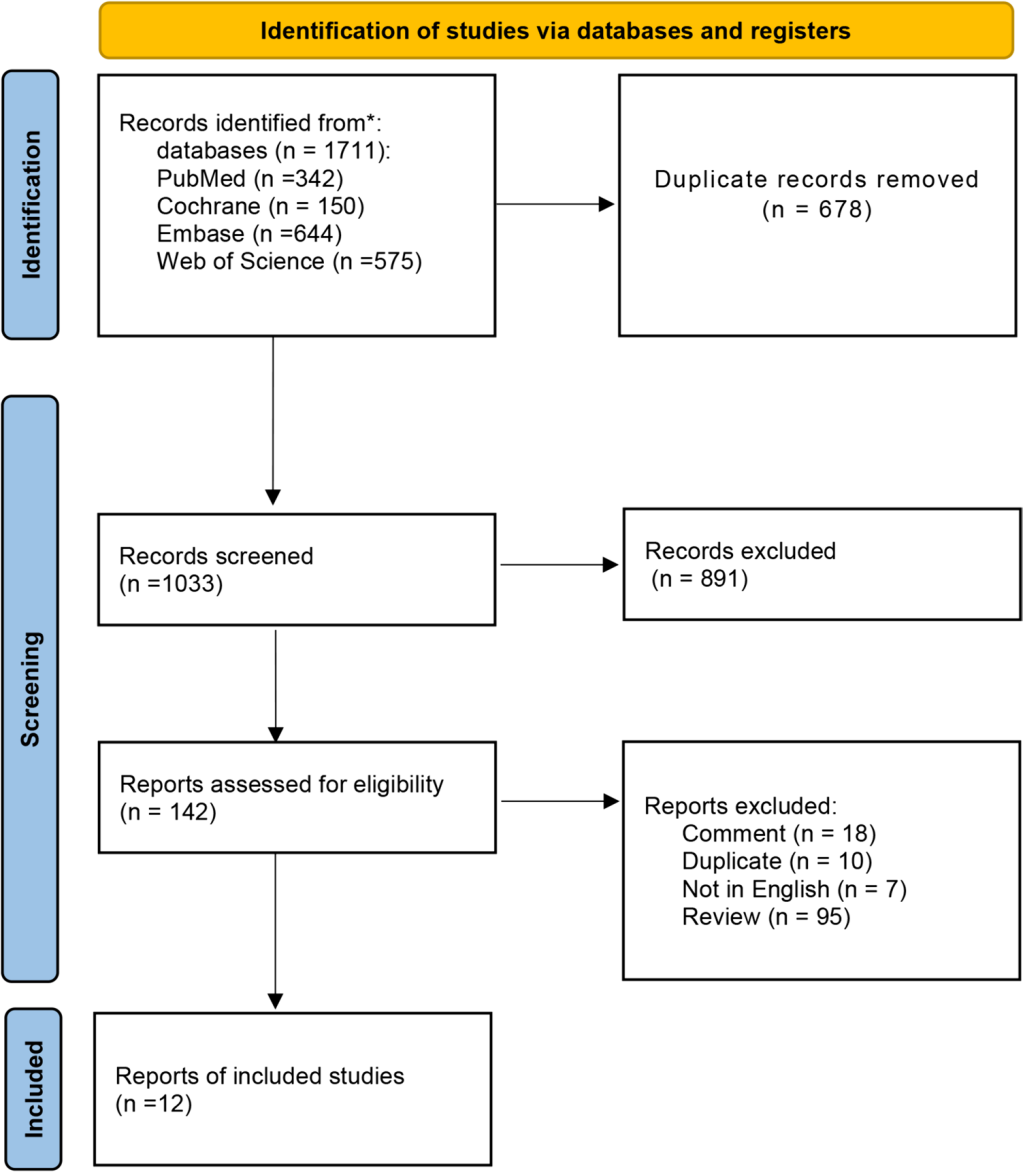
Flowchart of the systematic search and selection process

**Fig. 2:**
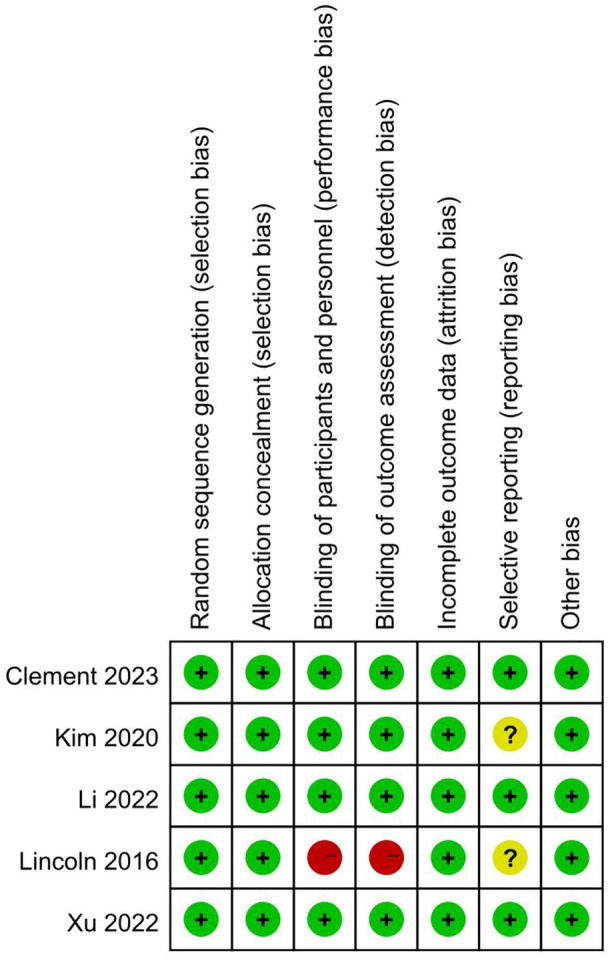
Risk of bias graph summary for randomized controlled trials

The process of literature retrieval and screening is displayed in detail in Fig. 1. Through systematic literature retrieval, 1,711 related articles were finally obtained from PubMed (*n* = 342), Embase (*n* = 644), Cochrane (*n* = 150) and Web of Science (*n* = 575). Then 1,033 titles and abstracts of studies were screened after deleting duplicates. Finally, 12 full-text articles were included, concerning 2,863 patients (1,449 RA-TKAs and 1,414 M-TKAs) for pooled analysis [11–13, 21–29]. Among them, five were RCTs [11, 12, 21–23] and seven were cohort studies [13, 24–29]. The specific features of all included studies are presented in Table 1. Quality evaluation of eligible cohort studies and RCTs is shown in Table S2 and Fig. 2, respectively.

**Table 1:** Baseline characteristics of the included studies

Authors	Study period	Country	Study design	Patients (n)	Median follow-up (months)
				RA-TKA/M-TKA	
Clement et al.	2019–2021	UK	Prospective	46/41	6
Xu et al.	2022	China	Prospective	37/35	3
Li et al.	2020–2021	China	Prospective	69/74	3
Kim et al.	2002–2008	South Korea	Prospective	674/674	156
Lincoln et al.	2015–2016	USA	Prospective	31/29	24
An et al.	2022	China	Retrospective	27/27	6
Kenanidis et al.	2020–2021	Greece	Retrospective	30/30	6
Khlopas et al.	2016–2018	USA	Retrospective	150/102	3
Jin et al.	2004–2007	South Korea	Retrospective	160/230	120
Babar et al.	2016–2017	UK	Retrospective	60/60	70
Moussa et al.	2019–2022	France	Retrospective	100/100	12
Kayhan et al.	2019–2020	Poland	Retrospective	70/46	24

### Change of Knee Society Score

**Fig. 3:**
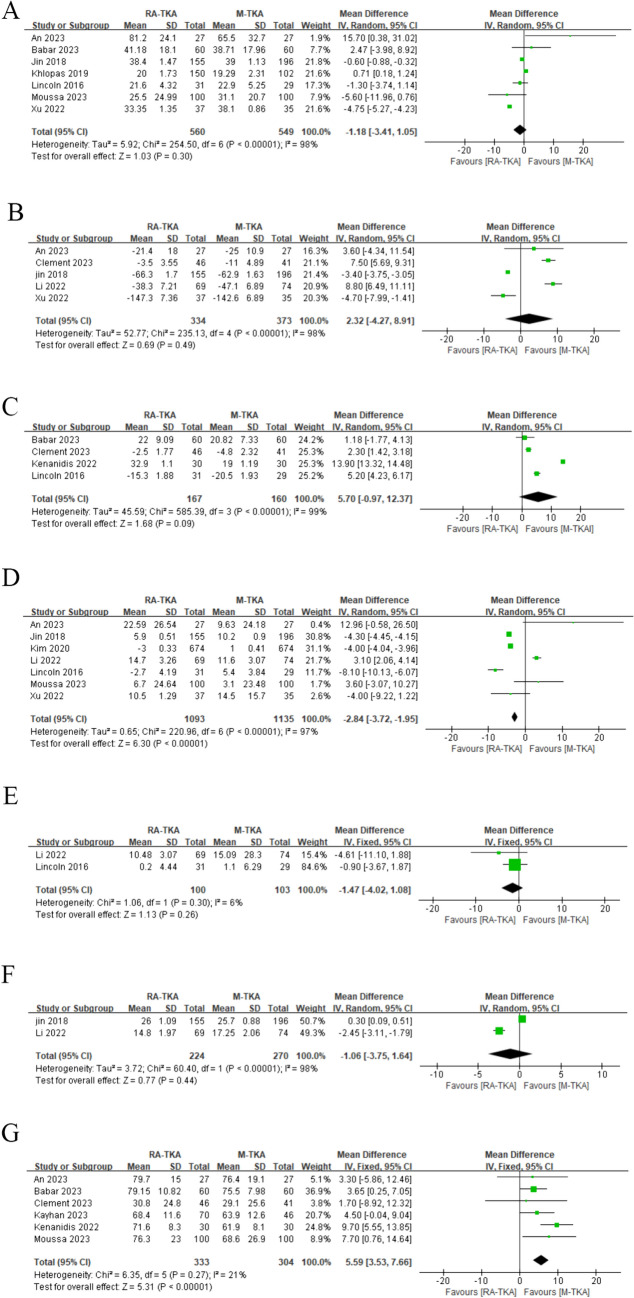
Forest plots of score: **a** KSS, **b** WOMAC, **c** OKS, **d** ROM, **e** SF*36, **f** HSS, **g** FJS

In the analysis of KSS improvement from preoperative to postoperative stages, the RA-TKA and M-TKA groups exhibited similar changes in KSS scores (WMD: −1.18; 95%CI: −3.41, 1.05: *p* = 0.30), with significant heterogeneity observed (*I*^2^ = 98%, *p* < 0.00001) (Fig. 3a). The subgroup analysis of > 6-month follow-up duration revealed a significantly lower KSS score in the RA-TKA group compared to the M-TKA group (WMD: −0.61; 95%CI: −0.89, −0.33: *p* < 0.00001) (Table 2).

**Table 2:** Subgroup analysis of RA-TKA versus M-TKA

Subgroup	KSS				Pain score				WOMAC				OKS				ROM			
	Study	MD [95%CI]	*P* value	*I*^2^	Study	MD [95%CI]	*P* value	*I*^2^	Study	MD [95%CI]	*P* value	*I*^2^	Study	MD [95%CI]	*P* value	*I*^2^	Study	MD [95%CI]	*P* value	*I*^2^
***Total***	7	-1.18 [-3.41, 1.05]	0.3	98%	4	1.51 [-0.34, 3.36]	0.11	93%	5	2.32 [-4.28, 8.93]	0.49	98%	4	5.70 [-0.97, 12.37]	0.09	99%	7	-2.84 [-3.72, -1.95]	< 0.00001	97%
***Study design***																				
RCT	2	-3.24 [-6.59, 0.11]	0.06	86%	2	1.79 [-4.48, 8.06]	0.58	93%	3	4.00 [-2.79, 10.79]	0.25	96%	2	3.74 [0.90, 6.58]	0.01	95%	4	-3.16 [-7.82, 1.51]	0.19	99%
Retrospective	5	0.02 [-1.33, 1.38]	0.97	85%	2	1.09 [-1.16, 3.34]	0.34	96%	2	-1.07 [-7.53, 5.40]	0.75	66%	2	7.63 [-4.84, 20.09]	0.23	99%	3	2.12 [-6.57, 10.82]	0.63	83%
***Follow-up***																				
> 6 months	5	-0.61 [-0.89, -0.33]	< 0.00001	49%	2	0.63 [-2.96, 4.21]	0.73	85%	1	-3.40 [-3.72, -3.08]	< 0.00001	NA	2	3.44 [-0.47, 7.35]	0.08	84%	4	-4.28 [-4.71, -3.85]	< 0.00001	91%
≤ 6 months	3	-0.42 [-5.56, 4.73]	0.87	99%	2	2.33 [-2.57, 7.23]	0.35	96%	4	3.93 [-1.98, 9.84]	0.19	94%%	2	8.10 [-3.26, 19.47]	0.16	100%	3	2.01 [-4.44, 8.46]	0.54	78%
***Region***																				
Asia	3	-1.58 [-5.57, 2.40]	0.44	99%	2	1.09 [-1.16, 3.34]	0.04	0%	4	0.93 [-6.19, 8.06]	0.8	97%					5	-2.24 [-3.17, -1.31]	< 0.00001	98%
Europe	2	-1.62 [-6.15, 2.91]	0.69	67%	1	4.90 [3.05, 6.75]	< 0.00001	NA					3	5.85 [-3.39, 15.08]	0.21	100%	1	3.60 [-3.07, 10.27]	0.29	NA
America	2	0.07 [-1.76, 1.91]	0.94	60%	1	-1.50 [-4.32, 1.32]	0.3	NA	1	7.50 [5.69, 9.31]	< 0.00001	NA	1	5.20 [4.23, 6.17]	< 0.00001	NA	1	-8.10 [-10.13, -6.07]	< 0.00001	NA

Subgroup	Operative duration				FFC angle				FTC angle				LFC angle				LTC angle			
	Study	MD [95%CI]	*P* value	*I*^2^	Study	MD [95%CI]	*P* value	*I*^2^	Study	MD [95%CI]	*P* value	*I*^2^	Study	MD [95%CI]	*P* value	*I*^2^	Study	MD [95%CI]	*P* value	*I*^2^
***Total***	3	26.03 [12.78, 39.28]	0.0001	86%	3	0.61 [-0.19, 1.42]	0.14	90%	3	0.13 [-0.99, 1.25]	0.82	94%	3	-0.80 [-3.73, 2.13]	0.59	99%	3	0.65 [-0.52, 1.81]	0.28	93%
***Study design***																				
RCT	1	7.93 [-4.62, 20.48]	0.22	NA	2	1.01 [0.81, 1.21]	< 0.00001	0%	2	0.51 [-0.56, 1.58]	0.35	88%	2	0.38 [-1.06, 1.83]	0.6	81%	2	1.06 [0.80, 1.32]	< 0.00001	45%
Retrospective	2	32.42 [23.60, 41.25]	< 0.00001	62%	1	-0.30 [-0.85, 0.25]	0.29	NA	1	-0.60 [-1.22, 0.02]	0.06	NA	1	-2.90 [-3.42, -2.38]	< 0.00001	NA	1	-0.60 [-1.16, -0.04]	0.03	NA
***Follow-up***																				
> 6 months	1	29.39 [28.47, 30.31]	< 0.00001	NA	2	0.38 [-0.90, 1.65]	0.56	95%	2	0.23 [-1.34, 1.80]	0.77	96%	2	-0.94 [-4.76, 2.88]	0.63	100%	2	0.22 [-1.35, 1.79]	0.78	96%
≤ 6 months	2	23.60 [-6.94, 54.15]	0.13	92%	1	1.10 [0.49, 1.71]	0.0004	NA	1	-0.10 [-0.82, 0.62]	0.78	NA	1	-0.50 [-1.75, 0.75]	0.43	NA	1	1.60 [0.77, 2.43]	0.0001	NA
***Region***																				
Asia	3	26.03 [12.78, 39.28]	0.0001	86%	3	0.61 [-0.19, 1.42]	0.14	90%	3	0.13 [-0.99, 1.25]	0.82	94%	3	-0.80 [-3.73, 2.13]	0.59	99%	3	0.65 [-0.52, 1.81]	0.28	93%
America																				
America																				

### Change of Western Ontario McMaster Universities Osteoarthritis Index

Five studies reported WOMAC. The pooled analysis indicated that RA-TKA and M-TKA groups demonstrated similar changes in WOMAC scores (MD: 2.32; 95%CI: -4.27, 8.91; *p* = 0.49), with salient heterogeneity observed (I^2^ = 98%, *p* < 0.00001) (Fig. 3b). The subgroup analysis of > 6-month follow-up duration unveiled a significantly lower WOMAC score in the RA-TKA group in contrast to the M-TKA group (WMD: −3.40; 95%CI: −3.72, −3.08; *p* < 0.00001) (Table 2).

### Change of Oxford Knee Score

**Fig. 4:**
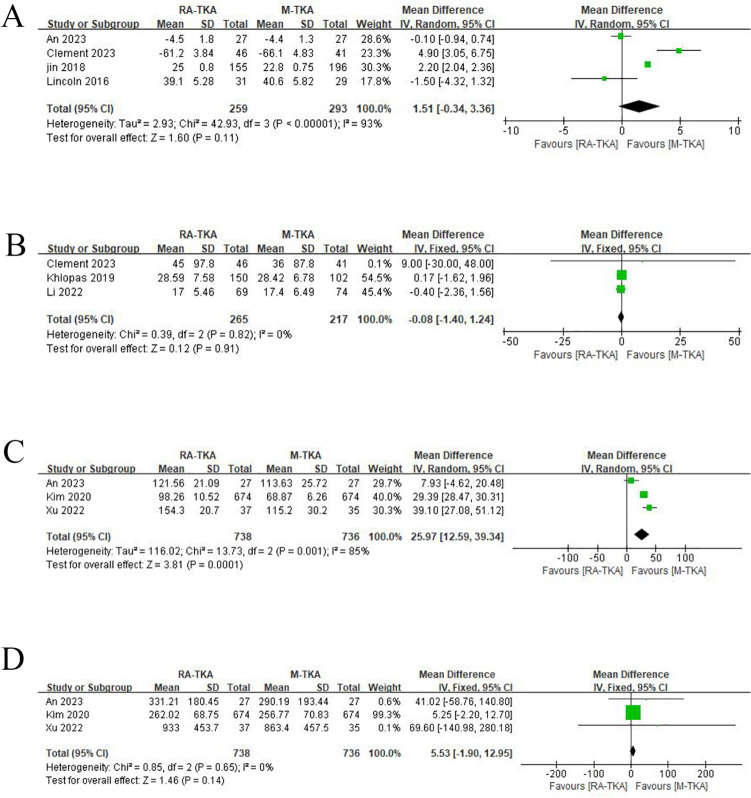
Sensitivity analysis of **a** KSS, **b** WOMAC, **c** OKS, **d** ROM, **e** pain score, **f** operative duration, **g** HKA, **h** FFC, **i** FTC, **j** LFC, **k** LTC

OKS was reported in four studies, which unraveled that the RA-TKA and M-TKA groups presented with similar OKS scores (WMD: 5.70; 95% CI: -0.97, 12.37; *p* = 0.09), with salient heterogeneity found (*I*^2^ = 100%, *p* < 0.00001) (Fig. 3). Sensitivity analysis showed that after excluding the research published by Lincon et al. in 2016 [23], the result changed from insignificant to significant, indicating instability of the index (Fig. 4c). The subgroup analysis of cohort studies, > 6-month follow-up duration, and European population revealed a significantly higher ROM score in the RA-TKA group compared to the M-TKA group (Table 2).

### Change of joint range of motion

Seven studies reported ROM. The comprehensive analysis suggested a significantly lower ROM score improvement in the RA-TKA group in contrast to the M-TKA group (WMD: −2.84; 95% CI: −3.72, −1.95; *p* = 0.00001), with significant heterogeneity observed (I^2^ = 98%, *p* < 0.00001) (Fig. 3d). Sensitivity analysis uncovered that after excluding the research published by Kim et al. in 2020 [12] or Jin et al. in 2018, the result changed from significant to insignificant, indicating instability of the index (Fig. 4d).

### Change of the 36-Item Short Form Health Survey score

The primary analysis demonstrated no statistically significant difference in postoperative SF-36 values between the RA-TKA group and the M-TKA group (WMD: −1.47; 95% CI: −4.02, 1.08; *p* = 0.26) (Fig. 3e), with significant heterogeneity (*I*^2^ = 6%, *p* < 0.30). Subgroup analysis similarly found no statistical difference.

### Change of hospital for special surgery

In the analysis of KSS improvement from preoperative to postoperative stages, the RA-TKA group and the M-TKA group indicated no statistical difference (WMD: −1.06; 95% CI: −3.75, 1.64; *p* = 0.44) (Fig. 3F) and notable heterogeneity was discovered (*I*^2^ = 98%, *p* < 0.00001).

### Forgotten Joint Score (FJS)

Six studies reported FJS. The pooled analysis unveiled a significantly higher FJS score in the RA-TKA group in contrast to the M-TKA group (WMD: 5.59; 95% CI: 3.53, 7.66; *p* < 0.0001) (Fig. 3g), with no significant heterogeneity (*I*^2^ = 21%, *p* = 0.27).

### Change of pain score

Four studies reported pain scores. The comprehensive analysis found no significant difference in pain scores between the RA-TKA and M-TKA groups (WMD: 1.51; 95% CI: -0.34, 3.36; *p* = 0.11). Sensitivity analysis showed that after excluding the research published by Linkon et al. in 2016, the result changed from insignificant to significant, indicating instability of the index (Fig. 4d).

### Patient satisfaction score

**Fig. 5:**
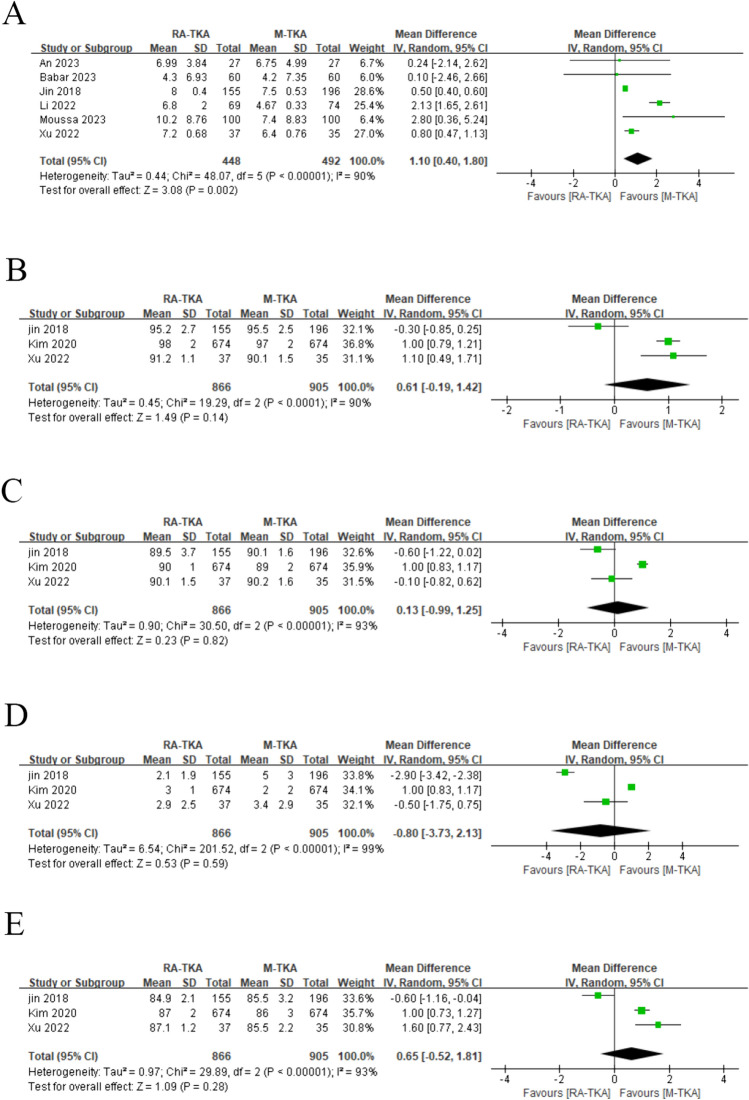
**a** Pain score, **b** patients' satisfactory score, **c** operative duration (min), **d** intraoperative blood loss (ml)

Three studies reported patient satisfaction scores. The pooled analysis showed that the RA-TKA and M-TKA groups exhibited similar patient satisfaction scores (WMD: -0.08; 95% CI: −1.40, 1.24; *p* = 0.91), with no salient heterogeneity (I2 = 0%, *p* = 0.82) (Fig. 5b).

### Operative duration

The operation time was reported in three studies. The comprehensive analysis found that the RA-TKA group exhibited a significantly longer operation time than the M-TKA group (WMD: 25.97; 95% CI: 12.59, 39.34; *p* = 0.0001), with significant heterogeneity (*I*^2^ = 85%, *p* = 0.001) (Fig. 5c). Sensitivity analysis uncovered that after excluding the research published by Kim et al. in 2020 or Xu et al. in 2022, the result changed from significant to insignificant, indicating instability of the index (Fig. 4f).

### Intraoperative blood loss

The comprehensive analysis revealed no statistically significant difference in intraoperative blood loss between the RA-TKA group and the M-TKA group (WMD: −5.53; 95%CI: −1.90, 12.95; *p* = 0.14), with no salient heterogeneity (*I*^2^ = 0%, *p* = 0.65) (Fig. 5d).

### Change of hip-knee-ankle angle

**Fig. 6:**
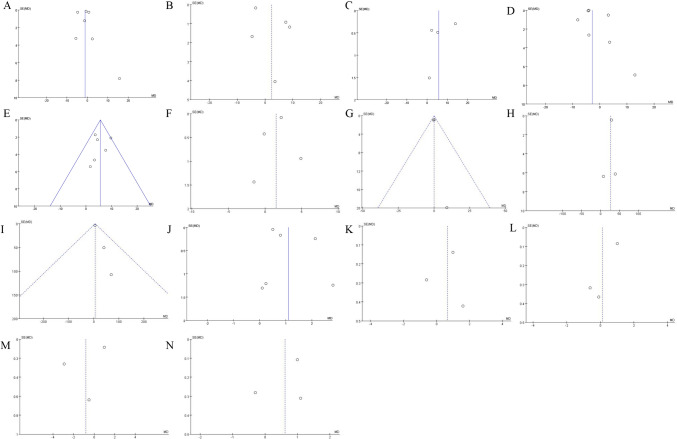
Radiographic findings: **a** HKA, **b** FFC, **c** FTC, **d** LFC, **e** LTC

HKA was reported in six studies. The comprehensive analysis suggested a significantly superior HKA score improvement in the RA-TKA group than that in the M-TKA group (WMD: 1.10; 95%CI: 0.40, 1.80: *p* = 0.002), with significant heterogeneity (I^2^ = 99%, *p* < 0.00001) (Fig. 6a).

### Frontal femoral component angle

Frontal femoral component (FFC) angle was reported in three studies. The comprehensive analysis found no clinical difference in the FFC angle between the RA-TKA group and the M-TKA group (WMD: 0.61; 95%CI: −0.19, 1.42; *p* = 0.14), with no salient heterogeneity (I^2^ = 90%, *p* < 0.0001) (Fig. 6b). Sensitivity analysis indicated that after excluding the research published by Jin et al. in 2018 [26], the result changed from insignificant to significant, indicating instability of the index (Fig. 4h). The subgroup analysis of RCT in a study revealed a significantly higher FFC score in the RA-TKA group compared to the M-TKA group (WMD: 1.01; 95%CI: 0.81, 0.21; *p* < 0.0001) (Table 2).

### Frontal tibia component angle

Three studies reported frontal tibia component (FTC) angle. Pooled analysis revealed no meaningful difference in FTC angle between the RA-TKA group FFC and the M-TKA group (WMD: 0.13; 95%CI: -0.99, 1.25; *p* = 0.82), and there was remarkable heterogeneity (*I*^2^ = 93%, *p* < 0.00001) (Fig. 6c).

### Lateral femoral component angle

Three studies reported lateral femoral component (LFC) angle. Pooled analysis revealed no significant difference in the LFC angle between the RA-TKA group FFC and the M-TKA group (WMD: −0.80; 95%CI: −3.73, 2.13; *p* = 0.59), and there was remarkable heterogeneity (*I*^2^ = 99%, *p* < 0.00001) (Fig. 6d). The cohort study subgroup analysis discovered a significantly lower LFC score in the RA-TKA group than in the M-TKA group (WMD: −2.90; 95%CI: −3.42, −2.38; *p* < 0.00001) (Table 2).

### Lateral tibia component angle

Three studies reported lateral tibia component (LTC) angle. Pooled analysis found no meaningful difference in LTC score between the RA-TKA group and the M-TKA group (WMD: 0.65; 95%CI: -0.52, 1.81; *p* = 0.28), and remarkable heterogeneity was noted (*I*^2^ = 93%, *p* < 0.00001) (Fig. 6f). Sensitivity analysis discovered that when the research published by Jin et al. in 2018 [26] was excluded, the result changed from insignificant to significant, indicating that the index was unstable (Fig. 4k). RCT subgroup analysis revealed a significantly higher FFC score in the RA-TKA group than in the M-TKA group (WMD: 1.06; 95%CI: 0.80, 1.31; *p* < 0.0001) (Table 2).

### Publication *bias*

**Fig. 7:**
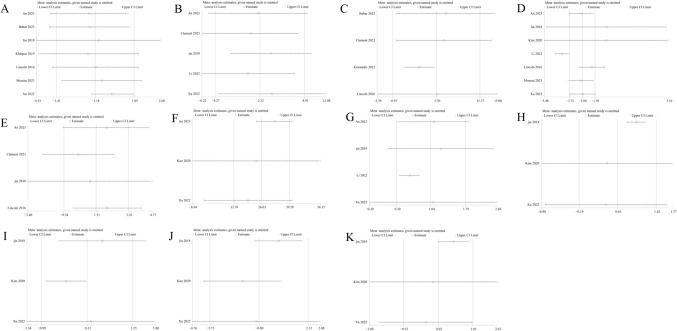
Funnel plots of **a** KSS, **b** WOMAC, **c** OKS, **d** ROM, **e** FJS, **f** pain score, **g** patients' satisfactory score, **h** operative duration, **i** intraoperative blood loss, **j** HKA, **k** FFC, **l** FTC, **m** LFC, **n** LTC

A visual assessment of funnel plots for all outcome measures (Fig. 7) was performed. Outcome measures showing potential publication bias were subjected to Egger’s test. No statistically significant publication bias was observed.

## Discussion

The most important findings of this meta-analysis are as follows: 1. The RA-TKA group demonstrated significantly better improvements in HKA angle and postoperative FJS score compared to the M-TKA group. 2. In contrast to the RA-TKA group, the operation time was shorter in the M-TKA group. Subgroup analysis of > 6-month follow-up results showed that the M-TKA group exhibited more significant improvements in KSS score and WOMAC score.

With the ultimate goal of building a stable, painless and long-lasting joint, M-TKA relies on surgical instruments to measure knee parameters, select a prosthesis and execute a surgical plan. Despite the proven efficacy and reproducibility of conventional knee arthroplasty, and ongoing innovations in prosthetics and surgical instruments, a notable number of patients remain dissatisfied with knee arthroplasty, attributed to various known and uncertain reasons [30, 31]. The primary reason often stems from the stringent standards of TKA for prosthesis placement, lower limb reconstruction and postoperative stability, while M-TKA struggles to consistently meet these criteria [32–34]. RA-TKA has been developed to eliminate potential inaccuracies in implant positioning and alignment, thus mitigating patient dissatisfaction. Numerous studies have unraveled that RA-TKA results in fewer outliers in component positioning, especially in the sagittal plane, irrespective of the knee alignment and balancing techniques employed. Surgeons may balance the knee more precisely with RA-TKA than with M-TKA [35]. However, it remains to be validated whether RA-TKA can improve postoperative function recovery and deliver superior clinical efficacy compared with M-TKA. On this basis, we conducted an up-to-date systematic review and meta-analysis.

The study has demonstrated that the RA-TKA group exhibited certain advantages in improving the HKA angle, thereby enhancing the alignment accuracy of the prosthesis and reducing the deviation of the lower limb force line from the neutral position. It has been reported that maintaining the (HKA) angle within a safe range of ± 3° can increase implant survival [36]. HKA angle is a crucial factor affecting the longevity of knee prosthesis. Abnormal HKA angle following TKA may result in prosthetic knee prosthesis dislocation, early postoperative prosthesis loosening, compromised functional recovery and heightened revision rate [37, 38]. Research by Mary K. Richardson et al. indicates that patients undergoing RA-TKA have a significantly closer-to-neutral postoperative HKA angle. In addition, compared to patients treated with conventional methods, fewer RA-TKA patients experience HKA angles outside the range of 0° ± 3° [39]. Byung Sun Choil et al. have demonstrated that the RA-TKA achieves superior accuracy and precision in femoral and tibial prosthesis placement compared to M-TKA, and all X-ray measurements were reproducible. These findings uncover that RA-TKA can enhance the accuracy and repeatability of component positioning and overall limb alignment [40–43]. It is worth noting that RA-TKA demonstrated the accuracy of prosthetic positioning and the enhancement of early patient-reported outcomes [35]. However, the correlation between accurate implant positioning and clinical outcomes remains contentious. A systematic review by Bensa et al. indicates that both procedures significantly improve patients’ symptoms, with no significant difference in clinical outcomes observed between RA-TKA and M-TKA, aligning with our own findings [44]. This raises questions about whether achieving a 180° alignment is universally “normal” and whether it should be the goal of TKA for all patients. Multiple studies have found that the HKA angle deviates from 0° in the general non-arthritic population. A study by Bellemans et al. has reported a varus angle of 1° in women and 2° in men based on a study of 250 healthy adults [45]. In addition, Almaavi et al. have reported a large variation in natural knee anatomy among 4884 CT scans of the knee, with only 5% of the general population exhibiting a natural neutral alignment (HKA angle: 0°) [46]. In most patients undergoing TKA, the knee may be compelled into an unnatural position, potentially contributing to the lack of corresponding clinical outcomes despite achieving better natural neutral alignment. Given the variability of coronal knee alignment in non-osteoarthritic knees and the wide variability of all coronal alignment parameters, the necessity is underscored for a more anatomically precise and individualized approach to knee arthroplasty [47].

The FJS is a joint-specific questionnaire designed to assess a patient’s ability to “forget" about a joint issue following joint treatment. It reflects not only the difference between “good” and “bad”, but also distinguishes between “good”, “very good” and “excellent” results [48]. In this study, the RA-TKA group was found to have a superior postoperative FJS in contrast to the M-TKA group. A study by Kafelov M et al. has unveiled that RA-TKA achieved a higher FJS at 1 year postoperatively compared with M-TKA [29]. Similarly, Kaanni et al. have reported that robot-assisted total knee arthroplasty is relevant to a statistically significant improvement in FJS compared to conventional total knee arthroplasty, although these differences fail to reach a minimal clinically important difference (MCID) at any follow-up interval [28]. Therefore, further comprehensive randomized controlled trials are needed for validation. The lower ceiling effect of the FJS allows monitoring of long-term outcomes, particularly in groups that show favorable outcomes following total joint arthroplasty. Measurable clinically significant differences between RA-TKA and M-TKA may be better demonstrated in future long-term studies [40].

The setup and registration of the robotic system in RA-TKA are unique and may lead to increased total operative time [49]. This study observed that the RA-TKA group had longer operative times than the M-TKA group, possibly due to the complexity of robotic surgical steps, operator inexperience, and the longer learning curve associated with RA-TKA. The study by Xu et al. has unraveled that in RA-TKA, a significant portion of operative time is devoted to tasks such as setup, femoral and tibial fixation, and alignment [22]. Longer operative time may elevate the rate of TKA infection, causing devastating consequences of TKA [50]. This is one of the disadvantages of RA-TKA, which can be improved by reducing the time allocated to non-surgical activities. As surgeons gain proficiency and RA-TKA techniques are refined, operative times may further decrease. The change of postoperative ROM in the M-TKA group was superior than that in the RA-TKA group. However, given the variability of the results, this result should be interpreted cautiously. In the subgroup analysis of the > 6-month follow-up, it was suggested that the improvement of KSS and WOMAC scores in the M-TKA group was significantly higher than that in the RA-TKA group, indicating potential advantages of long-term outcomes for the former. Short-term follow-up results were similar between the two groups. A recent meta-analysis has revealed that short-term patient-reported outcomes (KSS and WOMAC) are improved in the RA-TKA group compared with the conventional TKA group. However, these differences do not exceed the threshold for MCID, suggesting that they may not be clinically significant [35].

Limitations of the study: firstly, not all of the included studies were RCTs (5 RCTs and 4 retrospective cohort studies); secondly, due to limited data availability, no subgroup analysis was performed for different brands of robotic assistance systems. Considering the underlying factors, the results should be interpreted cautiously and may not be applied to all systems. As robotic systems evolve, new high-quality studies are warranted to assess the latest advancements in robotic systems. Thirdly, the study was constrained by a limited number of included studies and relatively small sample sizes, potentially limiting its representativeness for the broader population. Future studies with long-term follow-up are needed to establish more definitive conclusions regarding outcomes and benefits. Despite these limitations, our study incorporated recent analyses from both prospective and retrospective cohort studies. Sensitivity analysis and subgroup analysis were used to test the stability and publication bias of the results, providing a theoretical basis for large-scale prospective clinical trials and evidence support for clinical workers’ treatment choices.

## Conclusion

Our research results uncovered that the improvement of the HKA angle in the RA-TKA group was more significant than that in the M-TKA group. In terms of the operation time, improvement of ROM, KSS over 6 months and WOMAC score, M-TKA outperformed RA-TKA. The experimental follow-up time of this study was relatively short. Therefore, large-scale and well-designed clinical research with longer follow-up time is needed to comprehensively evaluate the advantages and disadvantages of the two surgical methods.

## Supplementary Information

Below is the link to the electronic supplementary material.

Supplementary file1 (DOCX 15 KB)Table S1 Detailed search strategy in three databasesTable S2 Quality evaluation of the eligible studies with Newcastle–Ottawa scale.

## Data Availability

The data that support the findings of this study are Pubmed at 1.https://doi.org/10.7507/1002-1892.202212016;2.https://doi.org/10.1302/0301-620x.105b9.bjj-2023-0006.r3;3.https://doi.org/10.1007/s00264-018-4231-1;4.https://doi.org/10.1007/s00590-022-03274-3;5.https://doi.org/10.1055/s-0039-1684014;6.https://doi.org/10.1097/corr.0000000000000916;7.https://doi.org/10.1016/j.jot.2021.12.004;8.https://doi.org/10.1007/s00167-016-4076-3;9.https://doi.org/10.1111/os.13323;10.https://doi.org/10.1007/s00167-023-07578-7;11.https://doi.org/10.1007/s00167-023-07609-3;12.https://doi.org/10.1007/s00167-023-07426-8.
