# Supplementary material for: Comparison of robotic-assisted total knee arthroplasty: an updated systematic review and meta-analysis
Source: J Robot Surg. 2024 Jul 25;18(1):292. doi: 10.1007/s11701-024-02045-y (PMC11272701; doi:10.1007/s11701-024-02045-y)
Supplement: Supplementary file 1 — Supplementary file1 (DOCX 15 KB)Table S1 Detailed search strategy in three databasesTable S2 Quality evaluation of the eligible studies with Newcastle–Ottawa scale. [file 11701_2024_2045_MOESM1_ESM.docx]

| Table S1. Detailed search strategy in three databases. | |
| --- | --- |
| Database | Search strategy |
| Pubmed | ((("Robotic Surgical Procedures"[Mesh]) OR ((((((((((((((((((((((((((Procedure, Robotic Surgical) OR (Procedures, Robotic Surgical)) OR (Robotic Surgical Procedure)) OR (Surgical Procedure, Robotic)) OR (Robot Surgery)) OR (Robot Surgeries)) OR (Surgery, Robot)) OR (Robot-Assisted Surgery)) OR (Robot Assisted Surgery)) OR (Robot-Assisted Surgeries)) OR (Surgery, Robot-Assisted)) OR (Robot-Enhanced Procedures)) OR (Procedure, Robot-Enhanced)) OR (Robot Enhanced Procedures)) OR (Robot-Enhanced Procedure)) OR (Surgical Procedures, Robotic)) OR (Robotic-Assisted Surgery)) OR (Robotic Assisted Surgery)) OR (Robotic-Assisted Surgeries)) OR (Surgery, Robotic-Assisted)) OR (Robot-Enhanced Surgery)) OR (Robot Enhanced Surgery)) OR (Robot-Enhanced Surgeries)) OR (Surgery, Robot-Enhanced)) OR (Robot)) OR (Robotic))) AND (("Arthroplasty, Replacement, Knee"[Mesh]) OR (((((((((((((((((((((((((((((((Arthroplasties, Replacement, Knee) OR (Arthroplasty, Knee Replacement)) OR (Knee Replacement Arthroplasties)) OR (Knee Replacement Arthroplasty)) OR (Replacement Arthroplasties, Knee)) OR (Knee Arthroplasty, Total)) OR (Arthroplasty, Total Knee)) OR (Total Knee Arthroplasty)) OR (Replacement, Total Knee)) OR (Total Knee Replacement)) OR (Knee Replacement, Total)) OR (Knee Arthroplasty)) OR (Arthroplasty, Knee)) OR (Arthroplasties, Knee Replacement)) OR (Replacement Arthroplasty, Knee)) OR (Arthroplasty, Replacement, Partial Knee)) OR (Unicompartmental Knee Arthroplasty)) OR (Arthroplasty, Unicompartmental Knee)) OR (Knee Arthroplasty, Unicompartmental)) OR (Unicondylar Knee Arthroplasty)) OR (Arthroplasty, Unicondylar Knee)) OR (Knee Arthroplasty, Unicondylar)) OR (Partial Knee Arthroplasty)) OR (Arthroplasty, Partial Knee)) OR (Knee Arthroplasty, Partial)) OR (Unicondylar Knee Replacement)) OR (Knee Replacement, Unicondylar)) OR (Partial Knee Replacement)) OR (Knee Replacement, Partial)) OR (Unicompartmental Knee Replacement)) OR (Knee Replacement, Unicompartmental)))) AND ((((((randomized controlled trial) OR (RCT)) OR (cohort)) OR (case-control)) OR (clinical trial)) OR (clinical study)) |
| Embase^*^ | (robotic AND surgical AND procedures OR (procedure, AND robotic AND surgical) OR (procedures, AND robotic AND surgical) OR (robotic AND surgical AND procedure) OR (surgical AND procedure, AND robotic) OR (robot AND surgery) OR (robot AND surgeries) OR (surgery, AND robot) OR ('robot assisted' AND surgery) OR (robot AND assisted AND surgery) OR ('robot assisted' AND surgeries) OR (surgery, AND 'robot assisted') OR ('robot enhanced' AND procedures) OR (procedure, AND 'robot enhanced') OR (robot AND enhanced AND procedures) OR ('robot enhanced' AND procedure) OR (surgical AND procedures, AND robotic) OR ('robotic assisted' AND surgery) OR (robotic AND assisted AND surgery) OR ('robotic assisted' AND surgeries) OR (surgery, AND 'robotic assisted') OR ('robot enhanced' AND surgery) OR (robot AND enhanced AND surgery) OR ('robot enhanced' AND surgeries) OR (surgery, AND 'robot enhanced') OR robot OR robotic) AND (arthroplasty, AND replacement, AND knee OR (arthroplasties, AND replacement, AND knee) OR (arthroplasty, AND knee AND replacement) OR (knee AND replacement AND arthroplasties) OR (knee AND replacement AND arthroplasty) OR (replacement AND arthroplasties, AND knee) OR (knee AND arthroplasty, AND total) OR (arthroplasty, AND total AND knee) OR (total AND knee AND arthroplasty) OR (replacement, AND total AND knee) OR (total AND knee AND replacement) OR (knee AND replacement, AND total) OR (knee AND arthroplasty) OR (arthroplasty, AND knee) OR (arthroplasties, AND knee AND replacement) OR (replacement AND arthroplasty, AND knee) OR (arthroplasty, AND replacement, AND partial AND knee) OR (unicompartmental AND knee AND arthroplasty) OR (arthroplasty, AND unicompartmental AND knee) OR (knee AND arthroplasty, AND unicompartmental) OR (unicondylar AND knee AND arthroplasty) OR (arthroplasty, AND unicondylar AND knee) OR (knee AND arthroplasty, AND unicondylar) OR (partial AND knee AND arthroplasty) OR (arthroplasty, AND partial AND knee) OR (knee AND arthroplasty, AND partial) OR (unicondylar AND knee AND replacement) OR (knee AND replacement, AND unicondylar) OR (partial AND knee AND replacement) OR (knee AND replacement, AND partial) OR (unicompartmental AND knee AND replacement) OR (knee AND replacement, AND unicompartmental)) AND (randomized AND controlled AND trial OR rct OR cohort OR 'case control' OR (clinical AND trial) OR (clinical AND study)) |
| Web of Science | (((Robotic Surgical Procedures) OR ((((((((((((((((((((((((((Procedure, Robotic Surgical) OR (Procedures, Robotic Surgical)) OR (Robotic Surgical Procedure)) OR (Surgical Procedure, Robotic)) OR (Robot Surgery)) OR (Robot Surgeries)) OR (Surgery, Robot)) OR (Robot-Assisted Surgery)) OR (Robot Assisted Surgery)) OR (Robot-Assisted Surgeries)) OR (Surgery, Robot-Assisted)) OR (Robot-Enhanced Procedures)) OR (Procedure, Robot-Enhanced)) OR (Robot Enhanced Procedures)) OR (Robot-Enhanced Procedure)) OR (Surgical Procedures, Robotic)) OR (Robotic-Assisted Surgery)) OR (Robotic Assisted Surgery)) OR (Robotic-Assisted Surgeries)) OR (Surgery, Robotic-Assisted)) OR (Robot-Enhanced Surgery)) OR (Robot Enhanced Surgery)) OR (Robot-Enhanced Surgeries)) OR (Surgery, Robot-Enhanced)) OR (Robot)) OR (Robotic))) AND ((Arthroplasty, Replacement, Knee) OR (((((((((((((((((((((((((((((((Arthroplasties, Replacement, Knee) OR (Arthroplasty, Knee Replacement)) OR (Knee Replacement Arthroplasties)) OR (Knee Replacement Arthroplasty)) OR (Replacement Arthroplasties, Knee)) OR (Knee Arthroplasty, Total)) OR (Arthroplasty, Total Knee)) OR (Total Knee Arthroplasty)) OR (Replacement, Total Knee)) OR (Total Knee Replacement)) OR (Knee Replacement, Total)) OR (Knee Arthroplasty)) OR (Arthroplasty, Knee)) OR (Arthroplasties, Knee Replacement)) OR (Replacement Arthroplasty, Knee)) OR (Arthroplasty, Replacement, Partial Knee)) OR (Unicompartmental Knee Arthroplasty)) OR (Arthroplasty, Unicompartmental Knee)) OR (Knee Arthroplasty, Unicompartmental)) OR (Unicondylar Knee Arthroplasty)) OR (Arthroplasty, Unicondylar Knee)) OR (Knee Arthroplasty, Unicondylar)) OR (Partial Knee Arthroplasty)) OR (Arthroplasty, Partial Knee)) OR (Knee Arthroplasty, Partial)) OR (Unicondylar Knee Replacement)) OR (Knee Replacement, Unicondylar)) OR (Partial Knee Replacement)) OR (Knee Replacement, Partial)) OR (Unicompartmental Knee Replacement)) OR (Knee Replacement, Unicompartmental)))) AND ((((((randomized controlled trial) OR (RCT)) OR (cohort)) OR (case-control)) OR (clinical trial)) OR (clinical study)) (Topic) and Preprint Citation Index (Exclude – Database) |
| Cochrane | (((Robotic Surgical Procedures) OR ((((((((((((((((((((((((((Procedure, Robotic Surgical) OR (Procedures, Robotic Surgical)) OR (Robotic Surgical Procedure)) OR (Surgical Procedure, Robotic)) OR (Robot Surgery)) OR (Robot Surgeries)) OR (Surgery, Robot)) OR (Robot-Assisted Surgery)) OR (Robot Assisted Surgery)) OR (Robot-Assisted Surgeries)) OR (Surgery, Robot-Assisted)) OR (Robot-Enhanced Procedures)) OR (Procedure, Robot-Enhanced)) OR (Robot Enhanced Procedures)) OR (Robot-Enhanced Procedure)) OR (Surgical Procedures, Robotic)) OR (Robotic-Assisted Surgery)) OR (Robotic Assisted Surgery)) OR (Robotic-Assisted Surgeries)) OR (Surgery, Robotic-Assisted)) OR (Robot-Enhanced Surgery)) OR (Robot Enhanced Surgery)) OR (Robot-Enhanced Surgeries)) OR (Surgery, Robot-Enhanced)) OR (Robot)) OR (Robotic))) AND ((Arthroplasty, Replacement, Knee) OR (((((((((((((((((((((((((((((((Arthroplasties, Replacement, Knee) OR (Arthroplasty, Knee Replacement)) OR (Knee Replacement Arthroplasties)) OR (Knee Replacement Arthroplasty)) OR (Replacement Arthroplasties, Knee)) OR (Knee Arthroplasty, Total)) OR (Arthroplasty, Total Knee)) OR (Total Knee Arthroplasty)) OR (Replacement, Total Knee)) OR (Total Knee Replacement)) OR (Knee Replacement, Total)) OR (Knee Arthroplasty)) OR (Arthroplasty, Knee)) OR (Arthroplasties, Knee Replacement)) OR (Replacement Arthroplasty, Knee)) OR (Arthroplasty, Replacement, Partial Knee)) OR (Unicompartmental Knee Arthroplasty)) OR (Arthroplasty, Unicompartmental Knee)) OR (Knee Arthroplasty, Unicompartmental)) OR (Unicondylar Knee Arthroplasty)) OR (Arthroplasty, Unicondylar Knee)) OR (Knee Arthroplasty, Unicondylar)) OR (Partial Knee Arthroplasty)) OR (Arthroplasty, Partial Knee)) OR (Knee Arthroplasty, Partial)) OR (Unicondylar Knee Replacement)) OR (Knee Replacement, Unicondylar)) OR (Partial Knee Replacement)) OR (Knee Replacement, Partial)) OR (Unicompartmental Knee Replacement)) OR (Knee Replacement, Unicompartmental)))) AND ((((((randomized controlled trial) OR (RCT)) OR (cohort)) OR (case-control)) OR (clinical trial)) OR (clinical study)) in All Text - (Word variations have been searched) |

Table S2: Quality evaluation of the eligible studies with Newcastle–Ottawa scale.

| Study | Selection | | | | Comparability | | Outcome | | |
| --- | --- | --- | --- | --- | --- | --- | --- | --- | --- |
|  | Representative-ness | Selection of  non-exposed | Ascertainment  of exposure | Outcome not present at start | Comparability on most important factors; Age、Gender、BMI | Comparability on other risk factors | Assessment of outcome | Long enough follow-up (median≥6 mths) | Adequacy  (completeness) of follow-up |
| AN Haoming | * | * | * | * | * | * | * | * | * |
| Kyu-Jin Cho | * | * | * | * | * | * | * | * | * |
| Anton Khlopas, MD | * | * | - | * | * | * | * | - | * |
| Eustathios Kenanidis | * | * | * | * | * | * | * | * | * |
| Babar Kayani | * | * | * | * | * | * | * | * | * |
| Moussa Kafelov | * | * | * | * | * | * | * | * | * |
| Kayhan Turan | * | * | * | * | * | * | * | * | * |

*indicates criterion met; - indicates significant of criterion not met.
